# African Ancestry Proportion Influences Ileal Gene Expression in Inflammatory Bowel Disease

**DOI:** 10.1016/j.jcmgh.2020.02.001

**Published:** 2020-02-10

**Authors:** A. Mo, C. Krishnakumar, D. Arafat, T. Dhere, H. Iskandar, A. Dodd, J. Prince, S. Kugathasan, G. Gibson

**Affiliations:** 1 Center for Integrative Genomics, Georgia Institute of Technology, Atlanta, Georgia; 2 Department of Pediatrics, Emory University School of Medicine and Children’s Healthcare of Atlanta, Atlanta, Georgia; 3 Division of Digestive Diseases, Emory University School of Medicine Adult Gastroenterology Program, Atlanta, Georgia

The influence of ancestry on inflammatory bowel disease (IBD) susceptibility has recently been examined via several large-scale genome-wide association studies,^1^^,^^2^ but the effects of ancestry-specific variation in risk and modifier gene expression on prognosis in IBD remain poorly characterized. It has been estimated fairly consistently that the prevalence of IBD is approximately 2 to 3 times greater in individuals of European versus African American (AA) descent,^3^ but the literature offers conflicting reports on complications and outcomes in AA versus European ancestry patients.^4^^,^^5^ AA individuals tend to be admixed, with approximately 80% African (YRI) and 20% European ancestry (CEU).^6^ Although genome-wide association studies have identified hundreds of variants associated with IBD risk, most research has been conducted on cohorts of exclusively European ancestry.^7^ Earlier studies on IBD risk variants, such as *NOD2*, concluded that mutations in AA individuals result from European admixture, and thus confer similar increases in risk.^8^ However, the largest genome-wide association studies to date of IBD in AA identified 2 novel African-specific loci associated with IBD, hinting at the existence of African-specific contributions to disease that remain to be elucidated.^2^ In this study, we evaluated ileal transcriptomic profiles of 154 individuals of AA and European ancestry with IBD, and characterized differential gene expression between populations. We then examined the effect of proportions of African and European ancestry in AA patients on gene expression, demonstrating that observed variation in gene expression between populations is heritable and not solely caused by environmental differences.

**Figure 1:**
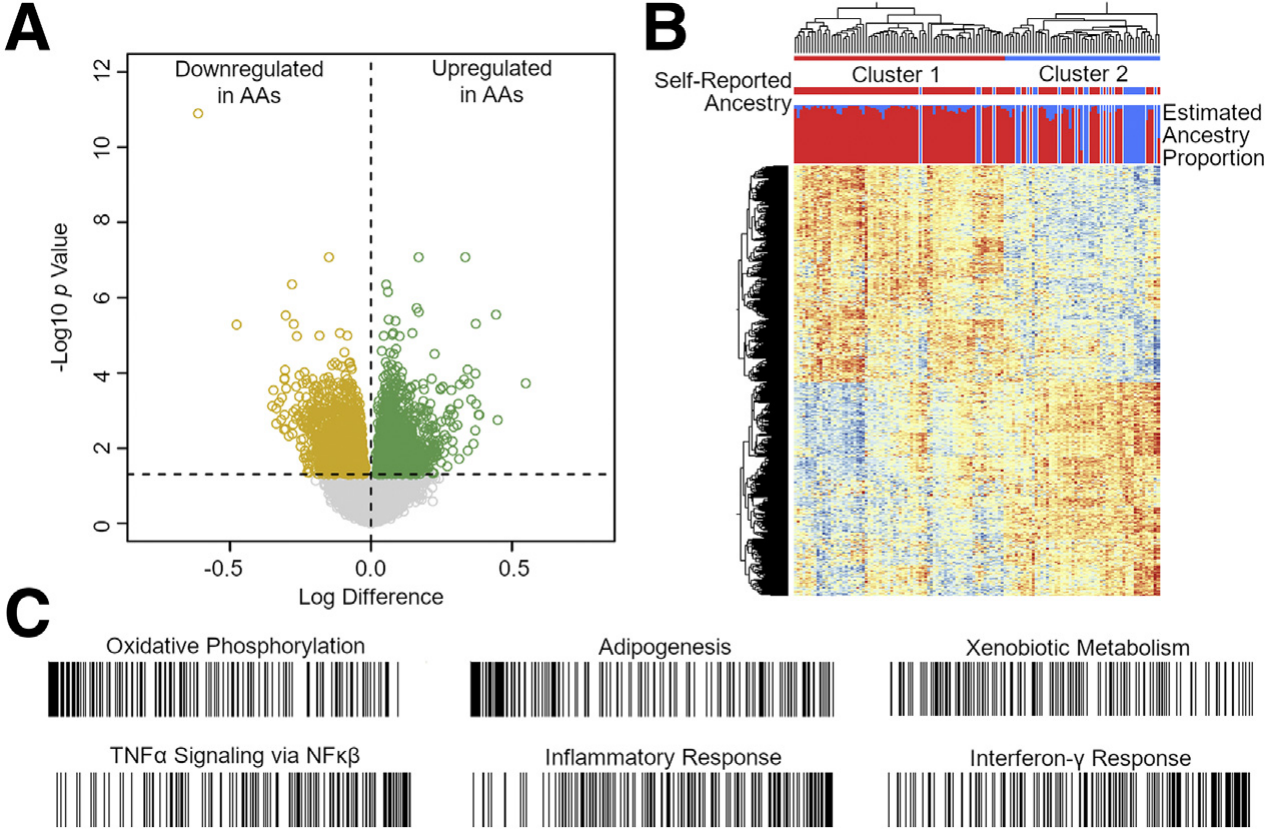
**Differential gene expression by ancestry.** (*A*) Volcano plot depicting log difference (x-axis) and –log10 *P* value (y-axis) for 14,392 genes between African (n = 104) and European (n = 25) ancestry patients. (*B*) Hierarchical clustering of 2705 genes differentially expressed at FDR <0.05 from *A*. *Top bar*: Individuals grouped into cluster 1 (*red*; n = 70 AA, 4 European) and cluster 2 (*blue*; n = 34 AA, 21 European). *Middle bar*: Self-reported AA (*red*) or European (*blue*) ancestry. *Bottom bar*: Estimated proportion of African (*red*) and European (*blue*) ancestry from supervised ADMIXTURE analysis. (*C*) Gene set enrichment analysis ranking plots. Each line represents a gene, whereas position from left to right represents ranking calculated by multiplying sign of fold change by inverse of FDR value. *Top row* shows bias toward downregulated genes in AA; *bottom row* shows bias toward upregulated genes. NF-kB, nuclear factor-kappa B; TNF, tumor necrosis factor.

We performed RNAseq of ileal biopsies sampled from control subjects (n = 25, no intestinal inflammation and normal histology) and 129 patients with ulcerative colitis (n = 36) and Crohn’s disease (n = 93). Differential gene expression analysis revealed 1360 upregulated and 1345 downregulated genes at an FDR cutoff of 0.05 in AA compared with European ancestry patients (Figure 1*A*). Hierarchical clustering based on these 2705 genes shows separation of transcriptomic profiles by ancestry into 2 clusters (Figure 1*B*). To explore functional pathways implicated by differentially expressed genes, we performed Gene Set Enrichment Analysis. Oxidative phosphorylation, adipogenesis, and xenobiotic metabolism were among the gene sets enriched for genes upregulated in AA, whereas tumor necrosis factor-α signaling, inflammatory response, and interferon-γ response were enriched in downregulated genes (Figure 1*C*, Supplementary Table 1). Each of these pathways is highly relevant to the development and progression of pathology in IBD, which highlights the importance of better understanding of genetic contributions to disease and possible personalized treatment options.

**Figure 2:**
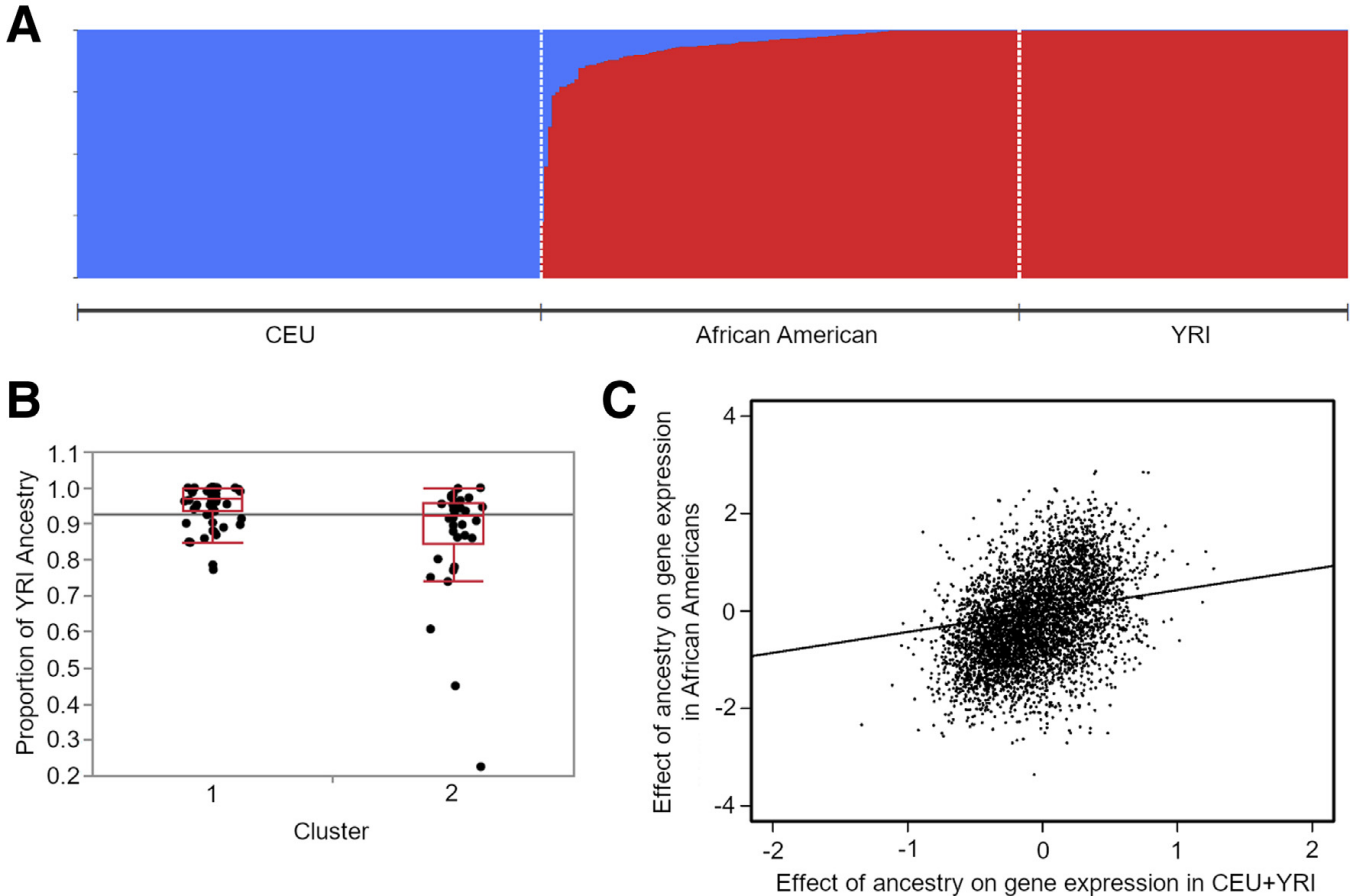
**Influence of ancestry proportions on gene expression.** (*A*) Proportions of ancestry assigned to AA. CEU population consists of 1000 Genomes Utah Northern and Western European ancestry individuals (n = 85) and European ancestry individuals from this study; YRI population consists of 1000 Genomes Yoruban individuals (n = 88). (*B*) Representative boxplot for analysis of variance between self-identified AA in cluster 1 (n = 70) and cluster 2 (n = 34) from Figure 1*B*. The y-axis represents proportion of YRI ancestry (*P* = 6×10^-5^). *Horizontal line* is grand mean. (*C*) Plot of regressed estimates of the effect of ancestry proportion on gene expression for the top 5000 most highly expressed genes, calculated in CEU versus YRI (x-axis) and within AA (y-axis). The slope of the correlation line is *c* = 0.43.

Following differential expression analyses, we sought to validate that the population-based variation in gene expression we observed is heritable and not solely attributable to environmental differences. By studying an admixed population, we can contrast the influences of European and African ancestry among individuals of mixed ancestry to the difference between the 2 predominant ancestry groups. Proportions of admixture in AA were estimated from common genotypes called from the RNAseq reads, using ADMIXTURE software^9^ with 1000 Genomes CEU and YRI individuals as reference European and African populations (Figure 2*A*). AA who group with European ancestry individuals in cluster 2 exhibit a highly significant (*P* = 6×10^-5^) trend of increased proportions of European admixture compared with AA grouping in cluster 1 (Figure 2*B*). We then applied methods introduced by Price et al^10^ to estimate the percentage of gene expression variation between populations that can be attributed to genetic effects. They argued that the slope of the regression of gene expression against ancestry proportion should be the same as the regression of the difference between the 2 predominant ancestry groups if the effect of ancestry is purely genetic, whereas if the 2 measures are uncorrelated it is purely environmental. The slope *c* of the 2 regressions assessed across all genes thus provides an estimate of the average heritability. By applying this method, we obtained *c* = 0.43, which we validated as statistically significant (*P* = .05) relative to 1000 permutations (Figure 2*C*, Supplementary Table 2). This value implies a substantial heritable component to differences in gene expression observed between AA and European ancestry IBD patients.

In summary, our study shows strong differential gene expression in key pathways based on African versus European ancestry. We further demonstrate that this variation in gene expression among populations can be partially attributed to heritable, genetic effects, rather than solely to differences in environmental factors. The pathways highlighted here are known to be critical in IBD pathogenesis, and elevation of inflammatory and tumor necrosis factor-α signaling seems to be consistent with evidence of worse prognosis in AA. Further investigation into ancestry-specific variation in disease is necessary for the development of personalized therapeutics.
