# Supplementary material for: African Ancestry Proportion Influences Ileal Gene Expression in Inflammatory Bowel Disease
Source: Cell Mol Gastroenterol Hepatol. 2020 Feb 10;10(1):203–5. doi: 10.1016/j.jcmgh.2020.02.001 (PMC7296223; doi:10.1016/j.jcmgh.2020.02.001)
Supplement: Supplementary Figure 1 and Supplementary Tables 1 and 2 [file mmc1.pdf]

Supplementary Table 1. **GSEA Enrichment Scores**

NAME	ES[1]	NES[2]	NOM p-val	FDR q-val
HALLMARK_MITOTIC_SPINDLE	-0.96953654	-2.738665	0.036809817	0.24540035
HALLMARK_TNFA_SIGNALING_VIA_NFKB	-0.78459835	-2.131841	0.061099797	1
HALLMARK_EPITHELIAL_MESENCHYMAL_TRANSITION	-0.7627758	-2.1261334	0.064718165	0.6822187
HALLMARK_INTERFERON_GAMMA_RESPONSE	-0.75405574	-2.0704424	0.06290673	0.5373365
HALLMARK_XENOBIOTIC_METABOLISM	0.7114698	1.7093651	0.14814815	0.8671167
HALLMARK_ANGIOGENESIS	-0.7054058	-1.7550359	0.047528517	0.33538553
HALLMARK_ESTROGEN_RESPONSE_EARLY	0.70333433	1.7096115	0.14570858	1
HALLMARK_COMPLEMENT	-0.6963707	-1.8607478	0.08898305	0.5260123
HALLMARK_ESTROGEN_RESPONSE_LATE	0.69636387	1.6763769	0.13644859	0.6136178
HALLMARK_OXIDATIVE_PHOSPHORYLATION	0.68012977	1.6174641	0.17375231	0.51181173
HALLMARK_KRAS_SIGNALING_UP	-0.6740996	-1.8120219	0.09544469	0.46603143
HALLMARK_INFLAMMATORY_RESPONSE	-0.66156536	-1.8029977	0.10107527	0.4036422
HALLMARK_HYPOXIA	-0.6506489	-1.7506601	0.11594203	0.3033227
HALLMARK_INTERFERON_ALPHA_RESPONSE	-0.6433157	-1.8028818	0.057539683	0.35318694
HALLMARK_MYC_TARGETS_V1	-0.63430864	-1.6890604	0.12035011	0.30162588
HALLMARK_IL6_JAK_STAT3_SIGNALING	-0.6031751	-1.66111	0.06584362	0.28307122
HALLMARK_SPERMATOGENESIS	-0.5676277	-1.557379	0.061099797	0.28568843
HALLMARK_HEDGEHOG_SIGNALING	-0.5453301	-1.3602403	0.13793103	0.29140854
HALLMARK_APOPTOSIS	-0.5123841	-1.419991	0.111561865	0.30515093
HALLMARK_PANCREAS_BETA_CELLS	0.50091386	1.2124683	0.23517382	0.6139624
HALLMARK_GLYCOLYSIS	0.5003059	1.1435319	0.256654	0.48104674
HALLMARK_MYOGENESIS	-0.4992916	-1.3265461	0.14197531	0.27315947
HALLMARK_ALLOGRAFT_REJECTION	-0.49219608	-1.3456522	0.12778905	0.28104284
HALLMARK_UV_RESPONSE_DN	-0.4788568	-1.3921219	0.09381663	0.29563344
HALLMARK_IL2_STAT5_SIGNALING	-0.4648526	-1.2194562	0.1826087	0.32190385
HALLMARK_PEROXISOME	0.45880708	1.1639911	0.21142857	0.5433961
HALLMARK_ADIPOGENESIS	0.42392758	1.0107353	0.29338843	0.5345546
HALLMARK_G2M_CHECKPOINT	-0.42264763	-1.1181176	0.18828452	0.39563367
HALLMARK_NOTCH_SIGNALING	-0.41112712	-1.0035685	0.41614908	0.5054806
HALLMARK_TGF_BETA_SIGNALING	-0.38988957	-1.0531788	0.34435797	0.45401844
HALLMARK_BILE_ACID_METABOLISM	0.37280008	0.92135537	0.38345864	0.5846342
HALLMARK_COAGULATION	-0.35666084	-0.96529174	0.36305732	0.55080724
HALLMARK_P53_PATHWAY	-0.35356408	-0.9617617	0.28817204	0.5333001
HALLMARK_E2F_TARGETS	-0.3495585	-0.89524615	0.31905782	0.5649946
HALLMARK_FATTY_ACID_METABOLISM	0.3491053	0.86028254	0.4208494	0.6140024
HALLMARK_PI3K_AKT_MTOR_SIGNALING	-0.34672815	-0.9346718	0.4032258	0.5377159
HALLMARK_APICAL_SURFACE	-0.34615594	-0.9051891	0.53373015	0.5665438
HALLMARK_CHOLESTEROL_HOMEOSTASIS	-0.34555647	-0.9500684	0.43452382	0.5313629
HALLMARK_ANDROGEN_RESPONSE	0.31548336	0.7782141	0.5708333	0.7003621
HALLMARK_REACTIVE_OXIGEN_SPECIES_PATHWAY	-0.3002477	-0.81362534	0.6687631	0.70644766
HALLMARK_WNT_BETA_CATENIN_SIGNALING	-0.29808396	-0.766772	0.7231076	0.73594713

HALLMARK_PROTEIN_SECRETION	0.29019544	0.6923406	0.65957445	0.80639565
HALLMARK_MYC_TARGETS_V2	-0.28258362	-0.77286494	0.69753087	0.74765736
HALLMARK_DNA_REPAIR	-0.27484703	-0.77776295	0.6492693	0.7620492
HALLMARK_UV_RESPONSE_UP	-0.26478726	-0.7135497	0.73100615	0.8207212
HALLMARK_MTORC1_SIGNALING	-0.25699028	-0.6710301	0.7731481	0.87532324
HALLMARK_KRAS_SIGNALING_DN	0.25573143	0.64437884	0.83943087	0.83299124
HALLMARK_APICAL_JUNCTION	-0.23773567	-0.65685195	0.8586724	0.87192327
HALLMARK_UNFOLDED_PROTEIN_RESPONSE	0.22921126	0.56387246	0.8996139	0.90224123
HALLMARK_HEME_METABOLISM	-0.22914925	-0.6322403	0.8836689	0.88378996

[1]Enrichment Score: Degree to which gene set is overrepresented at the top of bottom of the ranked list of genes.

[2]Normalized Enrichment Score: Enrichment score divided by the mean of enrichment scores against all permutations of the ranked list.

Supplemental Table 2. **Ancestry Proportion Permutation Test Summary Statistics**

**Statistic**	**Value**
Minimum	-0.619
1st Quartile	-0.227
Median	0.013
Mean	-0.005
3rd Quartile	0.210
Maximum	0.567
***P* Value of True Correlation[1]**	**0.05**

[1]Based on the ranking of true correlation (*c* =0.43) out of 1000 permutation tests.

Supplemental Figure 1. **Case-Control, Disease Subtype, and Medication Associations with DEGs**

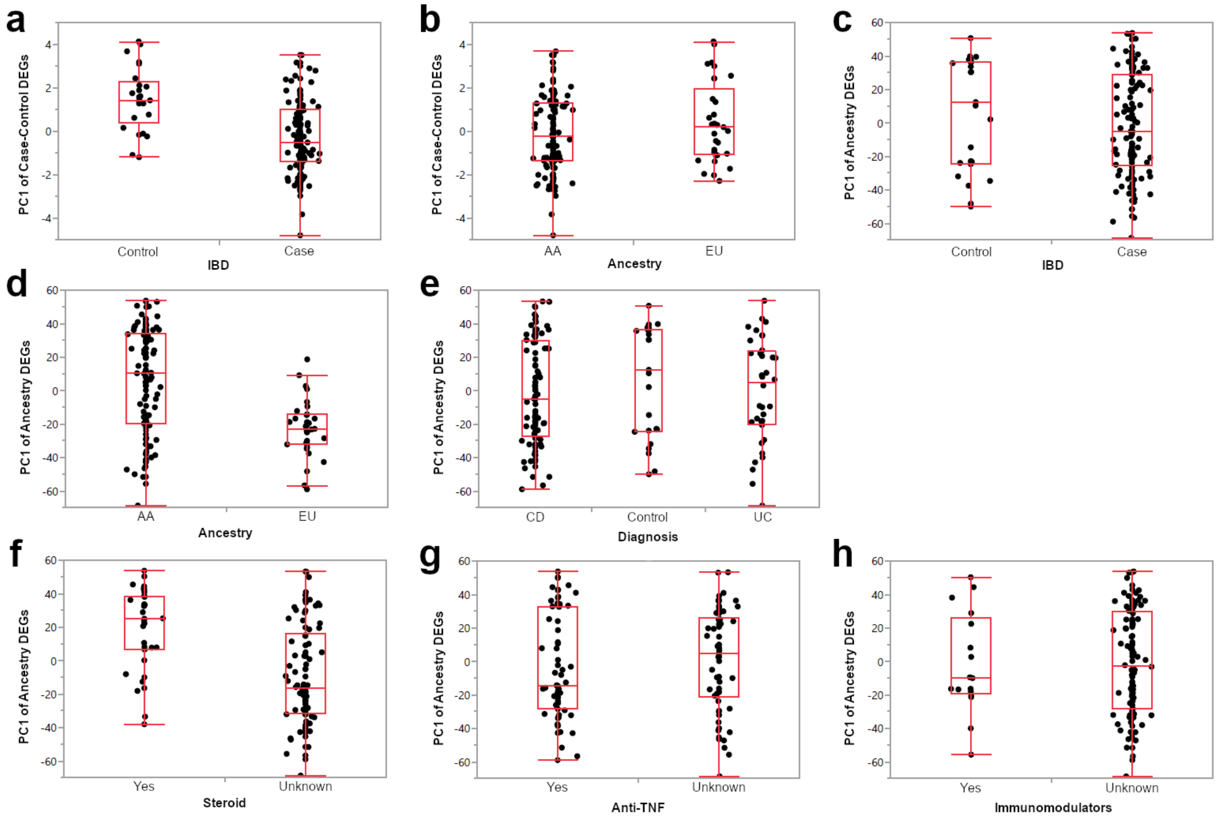

(a) First principal component (PC1) of genes differentially expressed between IBD cases and controls. $P = 1.42\times10^{-6}$ **(b)** PC1 of case-control DEGs in AA and European individuals. $P = 0.15$ **(c)** PC1 of genes differentially expressed between AA and European IBD patients in IBD cases and controls. $P = 0.27$ **(d)** First principal component of genes differentially expressed between AA and European IBD patients. $P = 1.38\times10^{-6}$ **(e)** PC1 of ancestry-related DEGs in controls and subtypes of IBD. $P = 0.54$ **(f)** PC1 of ancestry-related DEGs in patients known to be treated with steroids. $P = 9.22\times10^{-6}$; note that only AA patients received steroids **(g)** PC1 of ancestry-related DEGs in patients known to be treated with anti-TNF. $P = 0.44$ **(h)** PC1 of ancestry-related DEGs in patients known to be treated with immunomodulators. $P = 0.94$
