# Supplementary material for: African Ancestry Proportion Influences Ileal Gene Expression in Inflammatory Bowel Disease
Source: Cell Mol Gastroenterol Hepatol. 2020 Feb 10;10(1):203–5. doi: 10.1016/j.jcmgh.2020.02.001 (PMC7296223; doi:10.1016/j.jcmgh.2020.02.001)
Supplement: Supplementary Data [file mmc2.pdf]

Supplemental Methods – Mo et al (2020)

Cohort

In total, 129 patients and 25 controls were profiled for this study. Protocols included signed consent of all participants and/or assent of parents in the case of minors, and were approved by the IRBs of Emory University and Georgia Institute of Technology. Of the 154 total participants, 121 self-identified as African American and 33 identified as white. The cohort was evenly divided by gender, with 78 female participants and 76 male participants. Suspected IBD, chronic abdominal pain without known etiology, and unexplained weight loss were amongst the most common indications for colonoscopy to be performed in control individuals. Controls retained for this study had normal colonoscopy without inflammation, as well as normal histology verified through multiple pinch biopsies. The 129 patients in this study included 36 individuals with ulcerative colitis, of whom 28 were African American and 8 were European ancestry, and 93 individuals with Crohn's disease, of whom 76 were African American and 17 were European ancestry. The average age of onset amongst patients with UC and CD was approximately 14 years. Amongst the characterized CD patients, 18 had L1, 11 had L2, 53 had L3, and 1 had L1-L4 disease location; 54 had B1, 19 had B2, and 8 had B3 status. Amongst the characterized UC patients, 4 had E1 location, 6 had E2 location, and 25 had E3 disease location.

RNAseq processing and gene expression analysis

RNA was isolated from biopsies of the ileum for Lexogen 3' sequencing. Single end 75bp reads were trimmed for adapters with FastQC and Trim Galore, then mapped to human genome GrCh37 with the hisat2 aligner[1, 2]. The aligned reads were converted into read counts per gene using HTSeq[3]. The raw read counts were normalized with the edgeR R package implementation of trimmed mean of M-values normalization[4]. A combination of surrogate variable analysis (SVA) and supervised normalization (SNM) was then applied to remove batch effects and other confounding factors[5, 6]. First, expression of the sex-specific genes RPS4Y1, EIF1AY, DDX3Y, KDM5D, and XIST was checked to verify reported gender, resulting in the exclusion of 13 non-matching individuals. The SVA R package was then used to identify 6 surrogate variables which were then removed via supervised normalization in the SNM R package. Pairwise differential gene expression testing between African American and white IBD patients was then performed using the voom R package, which generated log fold change and Benjamini-Hochberg adjusted p-values for all genes[7]. Hierarchical clustering of the 2,705 genes differentially expressed at FDR < 0.05 was performed with the NMF R package. Gene set enrichment analysis was performed with GSEA, using pre-ranked mode on all 14,392 genes ranked by multiplying the sign of the fold change by the inverse of the Benjamini-Hochberg adjusted p-value[8]. Principal components of sets of differentially expressed genes were used to evaluate whether case-control status or therapeutic regimen explain the ancestry effects, as plotted in Supplementary Figure 1. With the exception of steroids, which were only given to a subset of AA patients, neither of these factors associate with ancestry.

Variant calling and calculation of ancestry proportion

The GATK Best Practices workflow for calling variants in RNAseq was followed to generate a VCF file of SNPs for individuals in this study[9]. VCF files for 1000 Genomes individuals belonging to either the CEU population (n=85) or YRI population (n=88) were extracted[10]. Both VCF files were merged, and quality control for genotyping rate was performed with PLINK, restricting the dataset to 12,819 variants[11]. Ancestry proportions for African American individuals were assigned using ADMIXTURE software in supervised mode, where 1000 Genomes CEU and white individuals from this study were provided as a known European population, and 1000 Genomes YRI individuals were provided as a known African population[12]. Plots of ancestry proportions were generated using the pophelper R package.

Calculation of heritable portion of gene expression variation

The calculation of the heritable portion of observed gene expression variation between populations in this study was based on methods first described by Price et al[13]. Individuals in this study were separated into CEU+YRI and African American population groups. 33 white individuals and 33 individuals with African ancestry proportions ~ 0.9999 were grouped into the CEU and YRI categories, while all other individuals were classified as African American. Gene expression across each gene was z-score normalized in the CEU+YRI group and African American group. Expression in the CEU+YRI group can be modeled as $e_{gs}=a_g\theta_s+v_{gs}$, where e_{gs} represents expression of gene g in individual s, a_g represents observed gene expression differences between CEU and YRI, θ_s denotes genome-wide African ancestry of either 0 or 1, and v_{gs}

represents residual effects. Then, $e_{gs}=ca_g\theta_s+v_{gs}$ for the African American group, where θ_s now ranges from 0 to 1 and c is a coefficient representing the extent to which a_g is heritable. An estimate of $a_{g,CEU+YRI}$ can be obtained by regressing e_{gs} against θ_s within the CEU+YRI group, and similarly an estimate of $a_{g,AA}$ can be obtained by regressing e_{gs} against θ_s within the African American group. An estimate of c can then be obtained by regressing the two estimates of a_g. The statistical significance of the estimated c was validated by testing the values of c obtained from 1000 sets of random permutations of African ancestry among African American individuals, then ranking the correlations. The permutation test yielded a p value of 0.05 for the c estimate based on true ancestry.

Supplemental References

1. Andrews S. FastQC: a quality control tool for high throughput sequence data, 2010.
2. Kim D, et al. Nat Methods 2015;12:357-60.
3. Anders S, et al. Bioinformatics 2015;31:166-9.
4. Robinson MD, et al. Bioinformatics 2010;26:139-40.
5. Leek JT, et al. Bioinformatics 2012;28:882-3.
6. Mecham BH, et al. Bioinformatics 2010;26:1308-15.
7. Law CW, et al. Genome Biology 2014;15:R29.
8. Subramanian A, et al. Proc Natl Acad Sci U S A 2005;102:15545-50.
9. McKenna A, et al. Genome Research 2010;20:1297-1303.
10. The Genomes Project C, et al. Nature 2015;526:68.
11. Purcell S, et al. Am J Hum Genet 2007;81:559-75.
12. Alexander DH, et al. BMC Bioinformatics 2011;12:246.
13. Price AL, et al. PLoS Genet 2008;4:e1000294.
